# 'Dysgenic fertility' is an ideological, not a scientific, concept. A Comment on: ‘Stability and change in male fertility patterns by cognitive ability across 32 birth cohorts’ (2023), by Bratsberg & Rogeberg

**DOI:** 10.1098/rsbl.2023.0390

**Published:** 2023-11-01

**Authors:** Rebecca Sear, Cathryn Townsend

**Affiliations:** ^1^ Population Health, London School of Hygiene and Tropical Medicine, London WC1E 7HT, UK; ^2^ Anthropology, Baylor University, Waco, TX 76798-7151, USA

**Keywords:** dysgenic fertility, eugenics, fertility, intelligence, ideology

## Abstract

Recently Bratsberg & Rogeberg (2023) presented an analysis in *Biology Letters* of how cognitive ability is associated with fertility in Norwegian men. Our concern relates to the theoretical framework of this paper. The analysis is framed around the concept of ‘dysgenic fertility’, which is treated throughout as a scientific theory, but ‘dysgenic fertility’ is not science, it is an ideological concept.

## 1. What is ‘dysgenic fertility’?

The concept of ‘dysgenic fertility’ stems from eugenic ideology, which was popularized in the ninteenth century by Charles Darwin's cousin Francis Galton [1,2]. Galton and other eugenicists believed that human populations can be ‘improved’ through selective reproduction; encouraging those with ‘desirable’ traits to have children while discouraging reproduction in those with ‘undesirable’ traits. ‘Improving’ the human population through selective breeding was referred to as ‘eugenics’. ‘Dysgenics’ was the term used as the antonym to eugenics, referring to the ‘degradation’ of the population through proliferation of ‘undesirable’ traits. ‘Dysgenic fertility’ was therefore a phrase used by eugenicists to indicate that people with ‘undesirable’ traits were having more children than those with ‘desirable’ traits. They believed this would lead to those ‘undesirable’ traits being selected for within the population, and so become more common.

Since Galton first coined the term eugenics, a common belief among eugenicists has been that higher cognitive ability, or ‘intelligence’, is a ‘desirable’ trait and a hallmark of inherently superior people. A negative association between cognitive ability and fertility is, therefore, an example of ‘dysgenic fertility’. Many eugenicists have used the claim that a negative association between cognitive ability and fertility exists in twentieth and twenty-first century human populations to argue for their preferred social policies, such as reduced social welfare. In the belief system of eugenicists, social welfare is often considered problematic because it encourages fertility among ‘undesirables’ [3–6].

The findings of Bratsberg & Rogeberg [7]'s paper in fact challenge the claims of eugenicists who posit that there is a negative association between cognitive ability and fertility, given they show a *positive* association between these traits in Norwegian men. They also provide evidence against the typical eugenicist claim that ‘social welfare policies raise the relative fertility of low-ability parents'. However, Bratsberg & Rogeberg have uncritically adopted the dysgenic framing of eugenicists throughout their paper: they simply conclude ‘there was no dysgenic male fertility’ in their population, without critiquing the concept of dysgenic fertility itself.

By uncritically adopting the framing of ‘dysgenic fertility’, they grant scientific legitimacy to three common eugenic beliefs or values:

1) the belief that higher cognitive ability is a ‘desirable’ trait,
2) the belief that it would be problematic for society if natural selection were to favour people with lower cognitive ability, and
3) the belief that cognitive tests measure a general, singular cognitive ability determined by genes and that therefore greater reproductive success of individuals who score lower on cognitive tests would effectively select for lower cognitive ability.

The first of these, the belief that higher cognitive ability is a ‘desirable’ trait, is a subjective value judgement, as there is no universally agreed definition of ‘desirable’. Some research finds greater cognitive ability is associated with life outcomes that are considered ‘good’ by many people, such as higher educational attainment, but it is also associated with life outcomes that are typically considered ‘bad’, such as Parkinson's disease and victimization at work [8–10]. Judgements can be made from these data about whether higher cognitive ability is desirable (for individuals) but they are value judgements, based on which outcomes are prized most highly by the person making that judgement.

Eugenicists’ belief systems take these value judgements further and assume that higher cognitive ability is ‘good’ for society (belief (2)). Measuring what is good or bad for society is even more subjective than measuring what is good or bad for individuals. This belief also gives a remarkable level of importance to this trait, such that people of higher cognitive ability are considered of more ‘worth’ to society. The goal of eugenics is to subvert the process of natural selection, not simply to observe it as a process, as ‘objective’ evolutionary science would do. If any evidence were to be found that natural selection is favouring those with lower cognitive ability (and the following paragraphs discuss why such evidence might be difficult to find), then an evolutionary scientist would conclude that those with lower cognitive ability are better adapted to contemporary environments than those with higher cognitive ability. Eugenicists instead conclude from their value system that if natural selection does not favour higher intelligence, then the situation is ‘bad’ for society and something must be done to intervene, even at the expense of violating human rights. The subjective values associated with eugenics should not be confused with objective knowledge (though we recognize that separating knowledge from values is not always easy).

Belief (3) has the appearance of a scientific hypothesis but would require a great deal more evidence to function in this way than Bratsberg & Rogeberg [7] provide. Bratsberg & Rogeberg do cite research claiming to find an association between polygenic scores for cognitive ability and fertility [11,12], but polygenic scores do not necessarily capture a causal relationship between genes and a complex trait such as cognitive ability, due to confounding factors such as cultural inheritance, assortative mating, dynastic effects and population stratification, [13–16]. Genetic and environmental entanglement has plagued behavioural genetic research on intelligence since long before the era of polygenic scores [13,17–19]. If the association between the identified genes and the trait is not in fact causal, then the eugenicists' assumption that selection acting on the associated genes would necessarily affect the frequency of the trait in descendent populations is invalid. ‘Cognitive ability’ is also typically measured in a culturally and context-specific way, prioritizing abilities that are developed in formal education, and is therefore a construct that does not necessarily have universal validity [20–23].

Even if we assume that (some measure of) cognitive ability can be accurately measured and that a causal relationship between genes and cognitive ability (as currently measured) exists, additional assumptions need to be made before it can be held that cognitive ability is being favoured or disfavoured by natural selection. For example, that associations between cognitive ability and fertility are consistent and stable over time, and that differences in polygenic scores cannot be accounted for by a neutral evolutionary model [24,25]. Belief (3) should therefore also be critically evaluated in any paper claiming to examine evidence that particular traits are being selected for, or against, in any human population.

## 2. If ‘dysgenic fertility’ is an ideological concept, why is it still being treated as science in the twenty-first century?

During the early twentieth century, eugenic ideology was widespread within science [26]. The increasing realization that eugenics cannot be scientifically justified, and that human rights abuses inevitably result from it, such as eugenic laws which made the sterilization of ‘undesirable’ individuals without their consent legal, and the Nazi regime exterminating ‘undesirables’ in horrifying numbers, led to a decline in the ideology within mainstream scientific literature. This decline was gradual; even after the Second World War, journals continued to publish on eugenic themes [27], and it may be that the terminology declined more than the ideology underpinning eugenics. The practice of eugenics certainly never went away: sterilizations have been performed without free and informed consent in many countries into the twenty-first century, typically targeted at marginalized groups [26,28–34].

Despite explicit discussion of eugenics having largely fallen out of the mainstream academic literature by the twenty-first century, many of its assumptions, such as belief in hereditary determinism (that the biological inheritance of traits or genes have a deterministic effect on life outcomes), have had remarkable longevity in the academic literature [26,30,35]. Pertinent to Bratsberg & Rogeberg's article is that, in addition, a small network of individuals has worked hard to keep eugenic ideology active on the fringes of the scientific literature [36–39]. As a recent example of this, the ‘London Conferences on Intelligence’ (LCI) discussed eugenic themes and were held at University College London (UCL) between 2014–17, in violation of UCL's regulations (see UCL's report: [40]). The first citation in Bratsberg & Rogeberg's paper, which they use to build their framework, has Michael Woodley as its senior author, who is part of this network. Woodley was an attendee of the LCI, and has published multiple works on ‘dysgenic fertility’, including two co-authored books, one titled *At our wits end: why we're becoming less intelligent and what it means for the future.* His ‘research’ promoting ideas of inevitable human hierarchies, a key principle underlying eugenics, was cited in the manifesto of the terrorist who carried out a fatal attack in Buffalo, USA, in 2022 (see [41]).

This eugenics network succeeds in publishing their ideology mostly in a small number of journals ‘friendly’ to this approach, including the Elsevier journal *Personality and Individual Differences* (the journal that published the Woodley article, used by Bratsberg & Rogeberg to build their argument around ‘dysgenic fertility’). These journal publications give the sheen of scientific respectability to eugenic ideology, and may deceive researchers unaware of the history of eugenics into believing it is legitimate science.

We are not suggesting that Bratsberg & Rogeberg deliberately intended to promote eugenic ideology with their paper, nor are we suggesting that their findings should not have been published; however, they have uncritically framed their analysis around an ideological concept, as if it were a scientific hypothesis. We are concerned to see explicit eugenic ideology appear in a highly respected biology journal and treated as if it were science. This re-emergence of explicit discussion of eugenics into mainstream academic journals likely reflects the reality that eugenic ideology continues to have a powerful influence in human society across multiple spheres, including academia, alongside the active promotion of this ideology by a small number of individuals deliberately trying to push eugenic ideas into the mainstream.

To avoid unwittingly reproducing eugenic ideology, we urge researchers, editors, and reviewers in the human sciences to make themselves familiar with the history of eugenics, and with how the scientific community continues to be exploited today by eugenicists (see e.g. [42,43]). Those working on research related to themes favoured by eugenicists, such as the process of natural selection in human populations, especially as it relates to cognitive ability, should also ensure they are well versed in relevant principles in evolutionary biology and genetics. A critical approach should be taken to research in the human sciences, ensuring it meets high standards of scientific rigour and that it is responsibly and sensitively communicated [44]. Due to the potential to cause harm, it is imperative that research in the human sciences is held to the highest scientific and ethical standards.

## Data Availability

This article has no additional data.

## References

[1] Jackson Jr JP, Weidman NM. 2006 Race, racism & science: social impact and interaction. New Brunswick, NJ: Rutgers University Press.

[2] Meyer MN et al. 2023 Wrestling with social and behavioral genomics: risks, potential benefits, and ethical responsibility. Hastings Cent. Rep. **53**, S2-S49. (10.1002/hast.1477) PMC10433733 37078667

[3] Kenny MG. 2004 Racial science in social context: John R. Baker on eugenics, race, and the public role of the scientist. Isis **95**, 394-419. (10.1086/428959) 15747772

[4] Jackson Jr JP. 2006 The origins of scientific racism. J. Blacks High. Edu. **50**, 66-79.

[5] Jackson Jr JP. 2023 Arthur Jensen, evolutionary biology, and racism. Hist. Psychol. **26**, 1-28. (10.1037/hop0000221) 35901381

[6] Wilmoth JR. 1997 Review of *Dysgenics: genetic deterioration in modern populations*, by R. Lynn. Popul. Dev. Rev. **23**, 664-666. (10.2307/2137584)

[7] Bratsberg B, Rogeberg O. 2023 Stability and change in male fertility patterns by cognitive ability across 32 birth cohorts. Biol. Lett. **19**, 20230172. (10.1098/rsbl.2023.0172) 37376853 PMC10300504

[8] Fardell C, Torén K, Schiöler L, Nissbrandt H, Åberg M. 2020 High IQ in early adulthood is associated with Parkinson's disease. J Parkinsons Dis. **10**, 1649-1656. (10.3233/JPD-202050) 32716321 PMC7683067

[9] Longinetti E, Zhan Y, Sata M, Larsson H, D'Onofrio BM, Iso H, Wirdefeldt K, Fang F. 2021 Heart rate, intelligence in adolescence, and Parkinson's disease later in life. Eur. J. Epidemiol. **36**, 1055-1064. (10.1007/s10654-021-00730-y) 33675447 PMC8542538

[10] Kim E, Glomb TM. 2010 Get smarty pants: cognitive ability, personality, and victimization. J. Appl. Psychol. **95**, 889-901. (10.1037/a0019985) 20718509

[11] Kong A et al. 2017 Selection against variants in the genome associated with educational attainment. Proc. Natl Acad. Sci. USA **114**, E727-E732. (10.1073/pnas.1612113114) 28096410 PMC5293043

[12] Hugh-Jones D, Abdellaoui A. 2022 Human capital mediates natural selection in contemporary humans. Behav. Genet. **52**, 205-234. (10.1007/s10519-022-10107-w) 35790706 PMC9463317

[13] Feldman MW, Ramachandran S. 2018 Missing compared to what? Revisiting heritability, genes and culture. Phil. Trans. R. Soc. B 373, 20170064. (10.1098/rstb.2017.0064) 29440529 PMC5812976

[14] Bird K. 2020 Still not in our genes: resisting the narrative around GWAS. *Science for the People* 23. See https://magazine.scienceforthepeople.org/vol23-3-bio-politics/genetic-basis-genome-wide-association-studies-risk/.

[15] Morris TT et al. 2020 Population phenomena inflate genetic associations of complex social traits. Sci. Adv. **6**, eaay0328. (10.1126/sciadv.aay0328) 32426451 PMC7159920

[16] Herzig AF, Clerget-Darpoux F, Génin E. 2022 The false dawn of polygenic risk scores for human disease prediction. J. Pers. Med. **12**, 1266. (10.3390/jpm12081266) 36013215 PMC9409868

[17] Bailey RC. 1997 Hereditarian scientific fallacies. Genetica **99**, 125-133. (10.1007/BF02259516) 9463068

[18] Turkheimer E. 2000 Three laws of behavior genetics and what they mean. Curr. Dir. Psychol. Sci. 9, 160-164. (10.1111/1467-8721.00084)

[19] Moore DS, Shenk D. 2017 The heritability fallacy. Wiley Interdiscip. Rev. Cogn. Sci. **8**, e1400. (10.1002/wcs.1400) 27906501

[20] Ceci S. 1996 Mismatches between intelligent performance and IQ. In On intelligence: a biological treatise on intellectual development (ed. S Ceci), pp. 29-44. Cambridge, MA: Harvard University Press.

[21] Richardson K. 2002 What IQ tests test. Theory Psychol. **12**, 283-314. (10.1177/0959354302012003012)

[22] Zuilkowski SS, McCoy DC, Serpell R, Matafwali B, Fink G. 2016 Dimensionality and the development of cognitive assessments for children in Sub-Saharan Africa. J. Cross-Cult. Psychol. **47**, 341-354. (10.1177/0022022115624155)

[23] Sear, R. 2022 ‘National IQ’ datasets do not provide accurate, unbiased or comparable measures of cognitive ability worldwide. PsyArXiv. (10.31234/osf.io/26vfb)

[24] Novembre J, Barton NH. 2018 Tread lightly interpreting polygenic tests of selection. Genetics **208**, 1351-1355. (10.1534/genetics.118.300786) 29618592 PMC5886544

[25] Rosenburg NA, Edge MD, Pritchard JK, Feldman MW. 2019 Interpreting polygenic scores, polygenic adaptation, and human phenotypic differences. Evol. Med. Public Health **2019**, 26-34. (10.1093/emph/eoy036) 30838127 PMC6393779

[26] Lombardo PA. 2018 The power of heredity and the relevance of eugenic history. Genet. Med. **20**, 1305-1311. (10.1038/s41436-018-0123-4) 30061627

[27] Bashford A. 2012 Epilogue: where did eugenics go? In The Oxford handbook of the history of eugenics (eds A Bashford, P Levine). Oxford, UK: Oxford University Press.

[28] Broberg G, Roll Hansen N. 2005 Eugenics and the welfare state: Norway, Sweden, Denmark, and Finland. East Lansing, MI: Michigan State University Press.

[29] Schoen J. 2005 Choice and coercion: birth control, sterilization, and abortion in public health and welfare. Chapel Hill, NC: University of North Carolina Press.

[30] Comfort N. 2012 The science of human perfection: how genes became the heart of American medicine. New Haven, CT: Yale University Press.

[31] Minna Stern A. 2016 Eugenic nation: faults and frontiers of better breeding in modern America. Oakland, CA: University of California Press.

[32] Bashford A, Levine P. 2012 The Oxford handbook of the history of eugenics. Oxford, UK: Oxford University Press.

[33] Appleman LI. 2018 Deviancy, dependency, and disability: the forgotten history of eugenics and mass incarceration. Duke Law J. **68**, 418-479. 30557924

[34] European Disability Forum. Report on forced sterilisation in the European Union. 2022. See https://www.edf-feph.org/publications/forced-sterilisation-eu/#:~:text=Forced%20sterilisation%20of%20persons%20with,widespread%20across%20Europe%20and%20worldwide.

[35] Selden S. 1999 Inheriting shame: the story of eugenics and racism in America. New York, NY: Teachers College Press.

[36] Panofsky A, Dasgupta K, Iturriaga N. 2021 How White nationalists mobilize genetics: from genetic ancestry and human biodiversity to counterscience and metapolitics. Am. J. Phys. Anthropol. **175**, 387-398. (10.1002/AJPA.24150) 32986847 PMC9909835

[37] Sear R. 2021 Demography and the rise, apparent fall, and resurgence of eugenics. Popul. Stud. **75**, 201-220. (10.1080/00324728.2021.2009013) 34902274

[38] Winston AS. 2020a Why mainstream research will not end scientific racism in psychology. Theory Psychol. **30**, 425-430. (10.1177/0959354320925176)

[39] Winston AS. 2020b Scientific racism and North American psychology. In Oxford research encyclopedia of psychology. New York, NY: Oxford University Press.

[40] UCL. 2018 UCL Investigation into London Conference on Intelligence. Draft report: Division of Psychology & Language Sciences. January 2018. See https://www.ucl.ac.uk/provost/sites/provost/files/lci_final_report_plus_appendices_web.pdf.

[41] Pronczuk M, Ryckewaert K. 2022 A racist researcher, exposed by a mass shooting. *New York Times, *9 June 2022.* *See https://www.nytimes.com/2022/06/09/world/europe/michael-woodley-buffalo-shooting.html.

[42] Saini A. 2019 Superior: the return of race science. London, UK: 4th Estate.

[43] Rutherford A. 2022 Control: the dark history and troubling present of eugenics. London, UK: W&N.

[44] Carlson J, Henn BM, Al-Hindi DR, Ramachandran S. 2022 Counter the weaponization of genetics research by extremists. Nature. **610**, 444-447. (10.1038/d41586-022-03252-z) 36261568

